# Improving introspection to inform free will regarding the choice by healthy individuals to use or not use cognitive enhancing drugs

**DOI:** 10.1186/1477-7517-6-10

**Published:** 2009-06-16

**Authors:** David S Thaler

**Affiliations:** 1 Raymond and Beverly Sackler Laboratory of Molecular Genetics and Informatics, Rockefeller University, New York, New York, USA

## Abstract

A commentary in Nature entitled "Towards responsible use of cognitive-enhancing drugs by the healthy" (Greely et al 2008 Nature 456: 702–705) offers an opportunity to move toward a humane societal appreciation of mind-altering drugs. Using cognitive enhancing drugs as an exemplar, this article presents a series of hypotheses concerning how an individual might learn optimal use. The essence of the proposal is that individuals can cultivate sensitivity to the effects of ever-smaller amounts of psychoactive drugs thereby making harm less likely and benign effects more probable. Four interrelated hypotheses are presented and briefly discussed. 1. Humans can learn to discriminate ever-smaller doses of at least some mind-altering drugs; a learning program can be designed or discovered that will have this outcome. 2. The skill to discriminate drugs and dose can be generalized, i.e. if learned with one drug a second one is easier and so on. 3. Cultivating this skill/knack would be beneficial in leading to choices informed by a more accurate sense of mind-body interactions. 4. From a philosophical point of view learning the effects of ever-smaller doses of psychoactive agents offers a novel path into and to transcend the objective/subjective barrier and the mind/body problem.

Whatever the fate of these specific hypotheses, discussion of cognitive enhancing drugs for healthy individuals has the potential to inspire innovative educational and public policy initiatives toward all types of mind-altering drugs and the people who use them.

## Commentary

A commentary in Nature advocates the availability of cognitive enhancing pharmaceuticals for healthy individuals [1].

Howard Gardner makes a strong [2] but controversial [3] case that human intelligence is most accurately described as a set of several distinct and independent intelligences. Independence implies there is no a priori reason to expect that a single pharmacology would enhance all forms of intelligence. Indeed a drug might enhance a particular type of intelligence while diminishing others.

Untoward effects are a fact of pharmacology. They are likely to be subtle, individual, difficult to anticipate, and important for drugs that modify the mind. New ways of thinking about the cost-benefit ratio are needed. Because the mind is subtle and individual, the cost-benefit ratio is likely to vary on an individual basis.

Cognitive enhancing drugs intended for healthy people ought to be drugs of choice. Informed free will is the ethical, and should be the practical, basis for decisions regarding their use. The cost-benefit ratio for these drugs comes down to a personal choice but one that can be informed.

Informing free will in this case means allowing and facilitating individual awareness of how a drug interacts with his/her mind. Effects and side effects are likely to be individual and need to be appreciated individually in order to facilitate choices. Both objective measures of intelligence(s) and subjective experience are important.

William James advocated introspection as an essential aspect of psychological research [4] including the effects of alcohol and nitrous oxide [5]. Introspection has largely been supplanted by objective and operational methods in most of psychology, but its resurgence would be especially timely in the context of intelligence enhancing drugs and harm reduction broadly interpreted. Introspection would seem an essential aspect of informing free will in the case of voluntary consumption of cognitive enhancing drugs by healthy individuals. The question becomes how to introspect optimally with respect to psychoactive drugs.

Introspection in this context is pragmatically defined as an individual's subjective process to seek understanding of their mind and body. Pharmacology is objective and the interface would have both aspects. Whatever someone does to try and sense the drug- whether it be sitting cross legged on a cushion with their eyes closed, monitoring their breath, or running up and down stairs- the efficacy of the process would be objective: What is the smallest amount of drug that they can discern and can they learn to increase their discernment?

Below are several related hypotheses concerning introspection and how it might be improved as a tool for healthy individuals to appreciate the effects of intelligence enhancing pharmaceuticals. The overall supposition is that the best choices concerning the voluntary consumption of mind-altering drugs will be made by those individuals who are best able to assess what particular drugs- and there is sure to be a wide choice of alternatives- are doing to their minds. The working hypotheses below are offered in a pragmatic spirit.

The core hypothesis is the optimistic expectation that the nervous system and consciousness have the potential to be self-referential in an accurate and positive manner. Undoubtedly, delusion and addiction are possible and known to be potential problems with respect to drugs that alter the mind. Having said that, it is reasonable to entertain the hypothesis that the mind can inform as well as fool itself. Introspection is a name for processes whereby the mind of an individual reflects on and assesses itself. If the optimistic hypothesis is accepted, then a further hypothesis is that all introspection is not equal and some modes of introspection are better than others. A further hypothesis is that enhancement and education of introspection is possible.

If it is possible to enhance pharmacological introspection, there is a field of research awaiting development to enhance and cultivate introspection directed toward understanding what drugs do to an individual's mind. More specific and guided forms of introspection and self observation could be proposed and would take the form of further specific hypotheses.

As an example I present the informal communication of a colleague regarding her experience with Insight Meditation [6] and the prescription drugs Xanax, Ativan, and Paxil. "The meditation practice was largely following my breath. I could feel my breath change when I was anxious. With the drug I was less anxious and this was reflected in my breathing. I lowered the dose of the drug over time and found that I was able to do the same thing to my breathing with less and less drug. Eventually I stopped taking the drug and am now able to simulate the drug's effect in my breathing and mind. However, there is a difference in my experience because when I was taking the drug I was much less aware of my body. It was as if my mind and body were separated. Now when I have an emotion I feel something in my body."

Although three drugs were mentioned she reported that it was primarily Paxil from which she reported quantitatively "weaning" herself with the aid of meditation practice. She herself suggested that her practice was akin to cultivating a placebo effect. A key distinction is that a placebo effect is based on deception [7,8] whereas her practice is the opposite: I.e. a critical examination of what the drugs do in her mind and a conscious mimicking of their effects as mediated through the breath.

Another informal respondent reported that he liked to ingest ca 50 mg methamphetamine once a month or so "Just to know how good- including how smart- you can feel". He apparently was not dependent on the drug but was using what he considered to be his drug-enhanced state as a benchmark, an internal role model, for how he wanted to feel and function.

These anecdotal examples suggest experimental methodologies that could lead to results that would either support or disprove the hypotheses. Volunteers would assimilate (ingest or via intravenous catheter) small and then increasing amounts of a mind-altering drug in a blinded manner. Each dose or placebo would be followed by a "sensing" period during which the subject would indicate whether or not they believe they sense the drug. A minimum dose that can be unambiguously sensed would be established. After a sufficient wash out period (hours or days) the test would be repeated. The question would be after e.g. 10 repetitions of the test would the experimental subject's threshold sensitivity for the drug be less than it was at the start? The subject would be encouraged and perhaps rewarded for every decrease in detection threshold. Numerous variants of the experimental protocol are conceivable and reasonable. For example, the first bolus might be large enough to assure a sensation and the threshold approached from above rather than from below. Training sessions might be unblinded in order for subjects to learn with certainty their internal sensation in response to ever-smaller doses of the drug.

Howard Gardner's distinct types of intelligence may or may not correspond to distinct classes of pharmacology. For example, Ritalin might improve an individual's ability to focus on a written exam and do math problems while diminishing his or her ability to draw on another sort of intelligence that enables him or her to do stand up comedy routines.

If the metaphor of a computer is applied to the mind then introspection becomes an algorithm subject to optimization. From the point of view of control theory as applied to the brain, the relationship between perception and response might place perception [9] and memory "outside" some sort of "central processor". By hypothesis, that placement is arbitrary and the brain has the potential to become skilled at perceiving changes in its internal state as it is altered by pharmacology.

The interface of introspection with objective pharmacological criteria is a bridge across the objective versus subjective dichotomy. Philosophical issues about the brain, mind and body are exciting [10] but the possibility of a new experimental program that can be refined through cycles of experiment and hypothesis formation seems uniquely valuable. The motivating hypotheses are that it is possible to improve self-understanding of pharmacological effects on one's brain, that this will involve cultivation of a generalized skill, and that the acquisition of this skill will be useful in the context of harm reduction. The experimental methods would involve training and testing of pharmacological sensitivity and its change over time.

The relationship between the hypothetical effect of sensitivity induced by introspection and the literature on sensitization [11] remains to be determined. An optimistic hypothesis is that acquiring an introspective skill for the effects of pharmacology will allow for desired and benign effects at lower doses. Detrimental effects including tolerance, addiction, anxiety and psychomotor agitation are differentially associated with higher doses and will thereby be diminished.

Consider the possibility of a drug that enhances introspection. Might there be drugs that increase the mind's perception of what drugs are doing to it? The hypothesized drug would increase the self-referential qualities of the nervous system. "Pay attention to attention." [12]

A specific hypothesis is that introspective education with respect to psychopharmacology will begin with training the mind to recognize diminishing quantities of a drug. By hypothesis, this is a general skill that can be learned whose enhancement will inform decision-making with regard to many drugs. Learning to become more sensitive is not expected in the context of pharmaceutical models that emphasize tolerance and addiction. On the other hand, in learning, increasing the subtlety of pattern apprehension is expected. Therefore it is a valid research question to ask which pharmaceuticals under what conditions fall under the addiction or under the learning model.

Can humans learn to discriminate the effects on their mind of smaller and smaller quantities of caffeine and/or alcohol? If one learns to better sense one change does that improve sensitivity to others? How improved and generalized could such skills of increased discrimination become? Introspection as outlined here is related to the current literature of drug discrimination (e.g [13]). These animal and human approaches are currently used to classify drugs as high or low potential for abuse. The cultivation of introspective skills that inform free will could become the central pillar of an education program based on a learning paradigm. The testable hypothesis is that a learning paradigm is possible and can be based on enhanced sensitivity to pharmacological effects.

Preceding the present and soon-arriving eras of intelligence enhancing drugs was the psychedelic era of "consciousness expanding" drugs. Clearly some things went wrong. The pitfalls of this era should be studied with the hope that they not be repeated. Three aspects deserve consideration: 1) Criminalization 2) Trivialization 3) Evangelical enthusiasm.

There are hints in the literature suggesting that some experienced users of psychedelics tended toward lower doses. An interesting article "Some notes concerning dosage levels of psychedelic compounds for psychotherapeutic experiences" [14] contains the following passage that implies learning the use of lower doses is possible, appropriate and often occurs without a specific instructional or theoretical background.

Use of small dosages with experienced subjects

This writer has often noted that experienced subjects tend to restrict themselves to a dosage level that they have found will induce a psychedelic experience. It is our opinion that this level is often unnecessarily high and we suggest that experienced individuals experiment with smaller dosages. It is common experience that a subject finds he needs a smaller amount of material to induce a psychedelic experience after he has had a few experiences with the larger dosage levels. However, individuals will often continue to use dosages of from 100 to 200 ug of LSD. It is hypothesized that as dosage is decreased, variables of the environment and the clarity of mind prior to the session become increasingly important. Consequently, prior to small dosage sessions, a period of meditation is highly useful to enable the individual to relax and to clear his consciousness of irrelevancies. Dosages as low as 10 ug to 25 ug LSD or one mg to two mg of psilocybin have been found to produce rather amazing states of expanded consciousness.

The discussion of cognitive enhancing drugs tends to assume that the effects will be temporary and that regular consumption will be required to sustain an effect. Anticipating that mode of use raises the specter of dependence and addiction. It is important to note that it is not the only model for how drugs that alter intelligence might act. Vaccines are an example of a pharmaceutical for which a single dose can yield benign consequences for a lifetime. Consider the possibility of a single dose of a drug that leads to life long consequences of increased intelligence.

To use or not use any particular drug to enhance the intelligence of healthy individuals ought to be an informed personal choice. An individual's appreciation of what the drugs do to his/her mind enhances the ability to choose wisely. Introspection has a place in informing free will and leading toward optimum outcomes of the opportunities afforded by the availability of intelligence enhancing drugs. Not all introspection is equal. A research program would characterize introspective states that are optimally sensitive to identifying the effects of drugs on the mind.

The experiences engendered by mind-altering drugs, as well as the motivations for their use, are strongly influenced by social and physical context [15,16]. There is not a firm line between the proposed use of legal intelligence enhancing drugs and the less legal use of drugs today. Stimulants in particular overlap with some of the cognitive enhancers. Functional users of methamphetamine vary widely with regard to their skill in titrating dosage to desired effect. Individual skill level at present may too often be regarded as if it is a fixed parameter of individual biology and/or socio-economic status rather than learnable. This inference seems unnecessarily pessimistic. The skill of riding a bicycle varies but is learned. The common situation with mind-altering drugs is as if users are popped onto their seats and pushed down a steep hill. That is an unforgiving way to learn. In fact it is not a situation for learning but rather for selection of certain preexisting characteristics. Scoring under non-learning conditions could explain why the skill level appears innate and fixed.

An analogous possibility for confusion between purely selective and learning-like processes arises in the study of bacterial mutation [17]. Immediately lethal selection allows only pre-existing mutants and in the absence of confounding factors leads to Luria-Delbruck kinetics[18]. On the other hand, conditions that are stressful but do not kill, at least in the short or medium term, allow bacteria opportunities to respond physiologically and to mutate genetically after the selection has been applied. The resulting adaptation under stress that is not immediately lethal can lead to properties that appear akin to "learning".

Learning could radically alter the already complex relationship of gene-environment interactions. New interpretations would become available to pharmacogenetics with regard to mind-altering drugs[19,20]. At an arguably extreme level of analogy, certain genes are key to the human ability to learn language[21] yet there is no apparent genetic specificity for one human language over another.

If the hypotheses in this article turn out correct, i.e. if skills of increased sensitivity to mind altering pharmacology can be cultivated, further work would be needed to explore their implications for drug use and abuse in different situations and contexts. A bridge between the laboratory and the library or the street would include the effect of context as well as internal state on the ability to judge pharmacology and dose.

This article proposes a series of four interrelated hypotheses that have the virtue of being subject to cycles of experimental inquiry leading to potential falsification and refinement: 1) Humans can learn to discriminate ever-smaller doses of at least some mind-altering drugs. 2) Discrimination skills generalize, i.e. once learned, discrimination will be easier with a second drug and so on. 3) This skill/knack would be beneficial in informing individual choices with more accurate sense of mind-body interactions. 4) From a philosophical point of view learning the effects of ever-smaller doses of psychoactive agents offers a novel path into and to transcend the objective/subjective barrier and the mind/body problem.

Whatever the fate of these specific hypotheses, discussion of cognitive enhancing drugs for healthy individuals has the potential to inspire innovative educational and public policy initiatives toward all types of mind-altering drugs and the people who use them.
